# Sex differences in a cohort of COVID-19 Italian patients hospitalized during the first and second pandemic waves

**DOI:** 10.1186/s13293-021-00386-z

**Published:** 2021-08-11

**Authors:** Virginia Quaresima, Cristina Scarpazza, Alessandra Sottini, Chiara Fiorini, Simona Signorini, Ottavia Maria Delmonte, Liana Signorini, Eugenia Quiros-Roldan, Luisa Imberti

**Affiliations:** 1 Centro di Ricerca Emato-oncologica AIL (CREA) and Diagnostic Department, ASST Spedali Civili of Brescia, Brescia, Italy; 2 Department of General Psychology, University of Padova, Padua, Italy; 3 University Department of Infectious and Tropical Diseases, University of Brescia, ASST Spedali Civili of Brescia, Brescia, Italy; 4 Immune Deficiency Genetics Section, Laboratory of Clinical Immunology and Microbiology (LCIM), National Institute of Allergy and Infectious Diseases (NIAID), National Institutes of Health (NIH), Bethesda, MD 20892-1456 USA

**Keywords:** COVID-19, Sex-related differences, Pandemic wave(s), Intensive care unit (ICU), SARS-CoV-2

## Abstract

**Background:**

Coronavirus disease 2019 (COVID-19) severity seems to be influenced by genetic background, sex, age, and presence of specific comorbidities. So far, little attention has been paid to sex-specific variations of demographic, clinical, and laboratory features of COVID-19 patients referred to the same hospital in the two consecutive pandemic waves.

**Methods:**

Demographic, clinical, and laboratory data were collected in 1000 COVID-19 patients (367 females and 633 males), 500 hospitalized in the first wave and 500 in the second one, at the ASST Spedali Civili of Brescia from March to December 2020. Statistical analyses have been employed to compare data obtained in females and males, taking into account their age, and during the first and second COVID-19 waves.

**Results:**

The mean age at the time of hospitalization was similar in females and males but was significantly higher for both in the second wave; the time elapsed from symptom onset to hospital admission did not differ between sexes in the two waves, and no correlation was observed between delayed hospital admission and length of hospitalization. The number of multi-symptomatic males was higher than that of females, and patients with a higher number of comorbidities were more frequently admitted to intensive care unit (ICU) and more frequently died. Older males remained in the ICU longer than females and showed a longer disease duration, mainly the first wave. The highest levels of white blood cells, neutrophils, C-reactive protein, and fibrinogen were significantly higher in males and in the first, and along with higher levels of D-dimer, ferritin, lactate dehydrogenase, and procalcitonin which were preferentially documented in patients requiring ICU or died. While the rate of death in ICU was higher in males, the overall death rate did not differ between the sexes; however, the deceased women were older.

**Conclusions:**

These data indicate that once patients were hospitalized, the risk of dying was similar between females and males. Therefore, future studies should aim at understanding the reasons why, for a given number of SARS-CoV-2 infections, fewer females develop the disease requiring hospitalization.

**Highlights:**

- Although the hospitalized males were significantly more, the similar number of hospitalizations of the > 75-year-old females and males could be due to the fact that in Brescia province, elderly women are about twice as many as men.
- Although males spent more days in the hospital, had a longer disease duration, developed a critical illness more frequently, and were admitted and died in the ICU more than females, the total rate of deaths among patients was not significantly different between sexes.
- Overall, the most frequent comorbidities were cardiovascular diseases, which were preferentially seen among patients hospitalized in the second wave; it is possible that the knowledge gained in the first wave concerning the association between certain comorbidities and worse disease evolution has guided the preferential hospitalization of patients with these predominant comorbidities.

**Supplementary Information:**

The online version contains supplementary material available at 10.1186/s13293-021-00386-z.

## Background

The ongoing severe acute respiratory syndrome coronavirus 2 (SARS-CoV-2) outbreak, originated in China in 2019, rapidly spread worldwide and the associated coronavirus disease 2019 (COVID-19) was declared a pandemic by the World Health Organization. COVID-19 has inexorably affected all countries, with increasing relevant social, economic, and health implications. In this global scenario, Italy was among the countries with the highest number of COVID-19 cases and deaths [1], and Brescia, with 102,628 confirmed cases of COVID-19 by 4 May 2021 [2], was one of the most affected Italian cities. As a consequence, the ASST Spedali Civili of Brescia, one of the largest hospitals in Italy, has assigned more than 800 beds to manage the COVID-19 emergency.

It is now well recognized that all individuals can be infected by SARS-CoV-2, albeit with different susceptibility, influenced by various factors, including the genetic background [3, 4] and age. Indeed, an increased infection rate is observed among females during childbearing age [5], while elders develop a more severe disease and are considered the main risk group for COVID-19 [1, 6]. In addition, a higher proportion of adverse outcomes and death occur in males [7] and the male bias in COVID-19 mortality has been demonstrated in nearly all countries, with a risk of death in males about 1.7 times higher than in females [8]. This is consistent with what was observed in animals and in the prior epidemics caused by SARS-CoV and Middle East Respiratory Syndrome CoV (MERS-CoV) [8]. These differences in male–female response to infection are not just limited to new coronaviruses, but it has been previously reported that male patients have higher viral loads for hepatitis B and human immunodeficiency viruses, while females generally mount a more robust immune response to vaccines, such as influenza vaccines [9].

The observed higher risk of death among males was present in all age groups and was associated with specific comorbidities, such as hypertension, cardiovascular disease, some chronic lung diseases, obesity, metabolic diseases, rates of tobacco smoking, and alcohol abuse that are more common among males than females [5, 10, 11]. Hence, differences in social and behavior gender-related factors may influence COVID-19 incidence and outcomes, though other biological mechanisms of male sex bias could affect the severity of COVID-19, particularly with respect to immune responses [12, 13].

While sex- and age-associated differences in COVID-19 patients have been previously investigated, most of the study published so far have involved small cohorts of patients, enrolled in a short period of time, or have been conducted by pooling results of multiple hospitals in the same region or from different countries, or by performing a meta-analysis [14–18]. In addition, not comprehensive clinical and laboratory parameters were analyzed in a single study. Therefore, the aim of our study was to compare the demographic, clinical, and laboratory features of a large number of COVID-19 patients referred to the same hospital in the two pandemic waves, and to investigate sex-specific difference of disease severity and mortality.

## Patients and methods

### Patients

The study cohort included 1000 patients hospitalized at the ASST Spedali Civili of Brescia from 23 March to 29 December 2020. It began with the announcement of the first cases of COVID-19 and involved the characterization of the first group of 500 patients; then the analysis was extended to other 500 subjects hospitalized during the second pandemic wave. Although there is no official date, the date of the COVID-19 second wave in Italy is considered 1 October 2020, when 2000 new daily cases were recorded for the first time in months [19].

The present study included patients who tested positive for SARS-CoV-2 by real-time polymerase chain reaction, had performed at least one laboratory test and were then hospitalized. Those with a positive laboratory test, but who required brief observation in the emergency room or who were dismissed from the hospital within a day, as well as those with doubtful positive swab or lacking a certain SARS-CoV-2 diagnosis, were excluded.

The demographic data, clinical characteristics, complications, treatment, clinical outcomes, and laboratory results were collected retrospectively.

In some cases, a comparison of patients’ age categories (< 45, 45–69, 60–74, and > 75 years old) and an analysis of different features occurred during the COVID-19 first wave and second wave were performed.

The classification of COVID-19 severity was assigned to each patient per the Diagnosis and Treatment Protocol for Novel Coronavirus pneumonia (trial version 7), released by the National Health Commission & State Administration of Traditional Chinese Medicine on 3 March 2020 [20].

The study was approved by the local Ethical Committee (Comitato Etico Provinciale, Brescia, Italy; protocols NP 4000–Studio CORONAlab and NP 4408–Studio CORONA follow-up).

### Laboratory testing

Blood samples were collected in microtubes containing ethylenediaminetetraacetic acid for the complete blood count, lithium heparin for biochemistry tests, and sodium citrate for hemostasis tests. Humoral and hemostasis parameters and complete blood count results were obtained by using automated CS-5100 (Siemens Healthcare s.r.l., Milan, Italy), COBAS 8000 (Roche, Basel, Switzerland), and XN 10 (Sysmex, Kobe, Japan) systems, respectively.

Reference values were those of the clinical laboratory of the ASST Spedali Civili of Brescia.

### Statistical analysis

Demographic and clinical data were analyzed by means of a chi square test for dichotomous variables, and by means of two independent samples *t* test or Mann-Whitney *U* test for continuous variables depending on whether data were or not normally distributed. The normal distribution of the data was assessed by means of the Kolmogorov-Smirnov (*d* test), where *d*> 0.20 means that the data are not normally distributed.

To evaluate the impact on sex and wave on time elapsed from symptom onset to hospital admission, a univariate ANOVA was performed using sex and wave as independent variables and days from symptom onset to hospital admission as dependent variables, and age as a covariate to rule out the influence of age on results. To assess the impact on sex and wave on dichotomous variables (e.g., comorbidities), logistic regressions were performed, using sex and wave as predictors and age as a covariate of no interest. Logistic regressions were also applied to assess the impact of main comorbidities and sex on clinical outcome (i.e., death). In this case, sex and comorbidity were included as predictors, death as a dependent variable, and age as a covariate of no interest.

Kaplan-Meier survival curves were performed: (1) on all 1000 patients, to investigate the influence of sex on length of hospitalization, using sex as factor, discharge (alive) from hospital as event and days from hospital admission to hospital discharge as main time variable; (2) on patients admitted to intensive care unit (ICU), to investigate the influence of sex on ICU length of stay, using sex as factor, discharge (alive) from ICU as event and days from ICU admission to ICU discharge as main time variable; (3) on the 813 patients with available data of symptom onset, to investigate the influence of sex on disease duration (defined as days from symptom onset to hospital discharge) using sex as factor, discharge (alive) as event and days from symptom onset to hospital discharge as the main time variable. Deaths were always censored. The difference in the curves was assessed through a log rank test.

To test the possible association between age and COVID-19 waves with (1) length of hospitalization, (2) ICU, length of stay, and (3) disease duration, a Cox-regression analysis was performed using sex, age, and waves as predictors and days as the main time variable. Hazard ratio (HR) and 95% confidence intervals (CI) are also reported.

## Results

### Characteristics of female and male patients hospitalized for COVID-19

Among 1000 patients included in this study, 367 (36.7%) were females and 633 (63.3%) males. The number of hospitalized females during the second wave was significantly higher than that in the first wave [199 (39.8%) vs 168 (33.6%); *P* = 0.042]. Within the patient’s cohort, only 57 (15.5%) females and 88 (13.9%) males were not Caucasian, similarly distributed in the two waves [78 (15.6%) in the first vs 67 (13.4%) in the second wave; *P* = 0.323].

**Fig. 1:**
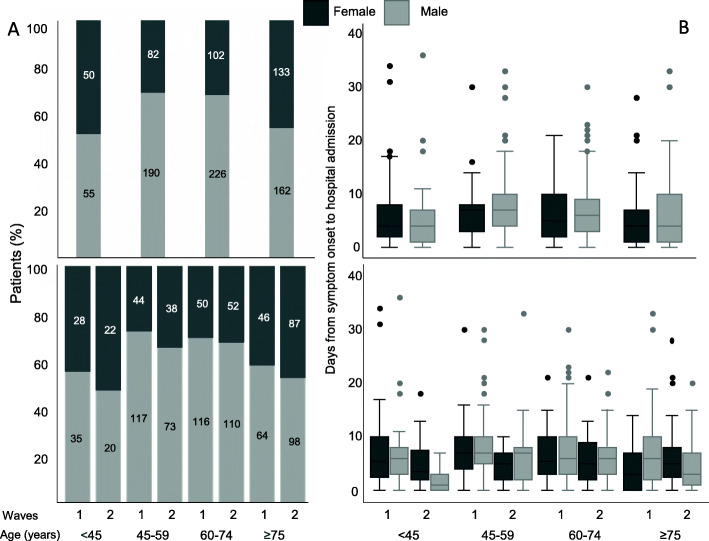
Sex distribution by age and hospitalization length of COVID-19 patients during the two waves **A** Total percentage and number (in the bars) of hospitalized females (dark gray) and males (light gray) divided according to the indicated age groups (top left panel) and age groups and waves (bottom left panel); 45–59 and 60–74 age groups, females vs males: chi-square = 24.72, *P* = 0.000 (top left panel), and 45–59 and 60–74 age groups, females vs males: chi-square = 4.403, *P* = 0.036 (bottom left panel). **B** Number of days between symptom onset and hospital admission in patient’s groups divided by age (top right panel-ANOVA: main effect sex *F* = 0.007, *P* = 0.933, main effect age *F* = 2.338, *P* = 0.07, interaction age × sex *F* = 1.364, *P* = 0.253) and by age and waves (bottom right panel-ANOVA: main effect sex *F* = 2.504, *P* = 0.807; main effect age *F* = 2.089, *P* = 0.100; main effect wave *F* = 14.395, *P* = 0.000; age × wave *F* = 1.664, *P* = 0.173; age × sex *F* = 1.937, *P* = 0.122; sex × wave *F* = 0.238, *P* = 0.626; sex × age × wave *F* = 1.390, *P* = 0.235)

The mean age + standard deviation at the time of hospital admission was 63.8 + 16.9 years, being similar in both sexes (65.01 + 9.3 in females vs 63.2 + 15.3 in males; *P* = 0.106), but significantly different between the first and the second wave (61.2 + 15.9 vs 67.5 + 17.9; *P* = 0.001). The rate of hospitalized females aged 45–59 and 60–74 years was significantly lower than that of age-matched males in both waves, while no differences were observed in the other two age groups (Fig. 1A).

The mean number of days from symptom onset to hospital admission was 6.5 + 5.7 and it was similar in both sexes (6.0 + 5.5 days in females vs 6.7 + 5.8 days in males; *P* =0.085) and age groups (Fig. 1B, top right panel). Patients who were admitted to the emergency room soon after the onset of symptoms were males aged < 45 years who were hospitalized in the second wave (Fig. 1B, bottom right panel). However, although ANOVA revealed a significant effect of the main variable wave (*F* = 12.01, *P* = 0.001), denoting a longer time from symptom onset to hospitalization in the first wave that in the second one (7.8 and 5.7 days, respectively), a non-significant effect has been found for the main variable sex (*F* = 2.11, *P* = 0.146). Similarly, given the sex × wave interaction not significant (*F* = 1.14, *P* = 0.286), the time elapsed from symptom onset to hospital admission did not differ between sexes in the two waves.

The condition which mainly led to hospitalization was the presence of interstitial pneumonia, which, however, was more frequently observed in males than in females (*P* = 0.002), both in the first and in the second wave, as resulted by the logistic regression analysis (sex × wave interaction; Supplementary Table 1). In addition, the percentage of multi-symptomatic males (showing 3 or more symptoms; 54.3%) was higher than that of females (45.2%) in both waves (effect of sex: *P* = 0.013, sex x wave interaction: *P* = 0.579, indicating that this effect does not change across waves). The main presenting symptom at the time of hospital admission was fever, which, in both waves, was more frequently mentioned by males than females (65.7% vs 56.9% respectively, *P* = 0.009). Cough was the second more frequent symptom, preferentially in the first wave in both sexes (37.2% vs 28.6% in the first and second wave, respectively, *P* = 0.013; sex × wave interaction: *P* = 0.545). The third more common sign was dyspnea, which was complained more by males than females and especially in the first wave (34.2% vs 28.3% in males and females, respectively, *P* = 0.068, 35% vs 29.2% in the first and second wave, respectively, *P* = 0.069; sex × wave interaction: *P* = 0.835). Loss of consciousness, which was reported significantly more in the first wave, and traumatic events were among the most frequent additional causes of hospitalizations in COVID-19 patients (Supplementary Table 1). These symptoms were not associated with a more severe outcome (data not shown).

The overall number of comorbidities was similar in females and males and no differences were observed in the number of COVID-19 patients with no comorbidities in both sexes, although this number was higher in the first wave. Likewise, there were no differences in patients with one comorbidity or simultaneously affected by 2, 3, or more concomitant pathologies (Supplementary Table 2).

The most common comorbidities identified in COVID-19 patients were those affecting cardiovascular and endocrine systems, with hypertension and diabetes being very common and present with similar incidence in the two sexes. Females were predominantly affected by psychiatric pathologies, autoimmune/immune-dysregulation disorders, and musculoskeletal and rheumatologic diseases, as well as by asthma. On the other hand, immunodeficiencies and infectious diseases were more common in males. In hospitalized patients, significant differences were observed in many specific comorbid conditions, as such cardiovascular diseases, solid malignancy, venous thromboembolism, and musculoskeletal disorders which were mainly reported in the second wave. On the contrary, a higher number of COVID-19 patients with thyroid diseases, neurological disorders, malignant and non-malignant hematologic diseases, immunodeficiency, and infectious diseases were hospitalized in the first wave. The logistic regression analysis, performed using the number of patients admitted to ICU as dependent variable and sex, wave, and comorbidity as independent variables as well as age as variable of non-interest, demonstrated that the number of patients admitted to ICU differs depending by the number of comorbidities. Out of 218 patients with 1 comorbidity and 164 with 2 comorbidities, 51 (23.3%) and 35 (21.3%) were admitted to ICU, respectively. Among these two groups of patients, there were more males than females. Lastly, out of 393 patients with more than 3 comorbidities, 59 (15%) were admitted to ICU.

### Clinical outcome of female and male patients hospitalized for COVID-19

**Fig. 2:**
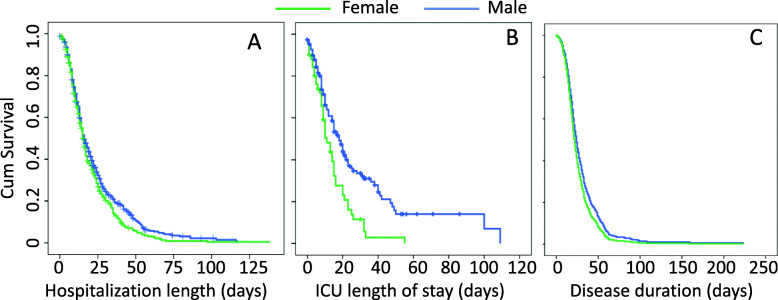
Kaplan–Meier curves of female and male COVID-19 patients. Kaplan-Meier curves indicate the days of hospitalization, from hospital admission to hospital discharge (**A**), the ICU length of stay (**B**), and the duration of the disease, starting from symptoms’ onset (**C**)

Kaplan-Meier-survival curve indicated that females and males differ in length of hospitalization (log rank *P* = 0.017), with males spending more days in hospital than females (mean 23.8 ± 0.9 days in males vs 20.2 ± 0.9 days in females; Fig. 2A). No correlation has been found between delayed hospital admission and length of hospitalization (*r* = − 0.022, *P* = 0.549), not even splitting patients into females and males (females *r* = 0.031; *P* = 0.151, and males *r* = − 0.066, *P* = 0.620).

When age and wave were entered as covariate in the Cox model, they were both significantly associated with hospitalization length (age: *P* = 0.001, HR = 0.981; CI = 0.977–0.986; wave: *P* = 0.001, HR = 0.790; CI = 0.688–0.907), denoting longer hospitalization for older individuals as well as longer hospitalization in the first wave compared with the second wave. However, despite the influence of age and waves on hospitalization length, the model having sex as predictor still remained highly significant (*P* = 0.004, HR = 1.232, CI = 1.071–1.418).

The percentage of hospitalized females with mild disease was significantly and consistently higher in both waves, while patients with asymptomatic, moderate, and severe COVID-19 were equally distributed in the two sexes (Table 1). The proportion of COVID-19 patients who required supplemental oxygen outside the ICU was similar in both sexes [196 (53.3%) in females vs 317 (50.1%) in males], but more female required low flow oxygenation.

**Table 1:** Clinical characteristics of hospitalized COVID-19 patients

	Females, *n* = 367	Males, *n* = 633	First wave, *n* = 500	Second wave, *n* = 500	Main effect of sex	Main effect of wave	Sex × wave interaction
*Severity*							
Asymptomatic	22 (6.0)	30 (4.7)	25 (5.0)	27 (5.4)	0.378	0.986	0.192
Mild	**39 (10.6)**	**33 (5.2)**	29 (5.8)	43 (8.6)	**0.001**	0.108	0.094
Moderate	110 (30.0)	169 (26.7)	127 (25.4)	152 (30.4)	0.273	0.175	0.219
Severe	60 (16.3)	117 (18.5)	**110 (22.0)**	**67 (13.4)**	0.69	**0.004**	0.085
Critical	**136 (37.1)**	**284 (44.9)**	209 (41.8)	211 (42.2)	**0.01**	0.54	0.363
*Types of oxygen supplementation*							
No oxygen support needed	109 (29.6)	153 (24.2)	124 (2.4)	138 (27.6)	0.055	0.687	**0.024**
Low flow cannula	**67 (18.2)**	**79 (12.5)**	**57 (11.4)**	**89 (17.8)**	**0.017**	**0.01**	0.461
High flow mask	71 (18.3)	136 (21.5)	**126 (25.2)**	**81 (16.2)**	0.626	**0.003**	0.283
cPAP/BiPAP	58 (15.8)	102 (15.1)	**63 (12.6)**	**97 (19.4)**	0.464	**0.001**	0.106
*ICU admission*							
Admission to ICU	**51 (13.9)**	**153 (24.2)**	**120 (24.0)**	**84 (16.8)**	**0.000**	**0.019**	0.967
Age of patients admitted to ICU^a^	**56.9 (16.9)**	**63.0 (12.3)**	**59.3 (13.7)**	**64.5 (13.6)**	**0.003**	**0.024**	0.665
*Outcome*							
Deaths within the whole dataset	51 (13.89)	96 (15.2)	71 (14.2)	76 (15.2)	0.462	0.331	0.099
Deaths within patients admitted to ICU	**6/51 (11.7)**	**53/153 (34.6)**	37/120 (30.8)	22/84 (26.2)	**0.004**	0.969	0.607

Row numbers (percentages) and statistical significances, indicated as *P* value (chi-square test), are reported for females and males, first and second waves

^a^For the variable “age of patients admitted to ICU,” the numbers denote mean (standard deviation). The normal distribution of this variable was tested using the Kolmogorov-Smirnov test

The three columns at the right-hand side report the results of the logistic regressions (or univariate ANOVA in the case of the continuous variable) using sex and wave as predictors and age as a covariate of no interest. A not significant sex × wave interaction denotes that the percentages of males and females for each condition do not differ across waves. Statistically significant results are reported in bold

*BiPAP* bilevel positive airway pressure, *cPAP* continuous positive airway pressure, *ICU* intensive care unit

Males developed critical illness more frequently as also reflected by the higher number of males who were admitted to ICU. Indeed, among the total number of patients managed in ICU (204, 20.4%), the rate of males was significantly higher than that of females. Moreover, significantly more patients were admitted to ICU in the first wave (Table 1).

The not significant sex × wave interaction at the logistic regression analysis denotes that the percentages of males and females for each condition described in Table 1 did not differ across waves. The only significant sex × wave interaction was observed for the variable “no oxygen support needed” and indicates that, only during the first wave, the number of females who did not need oxygen therapy was higher than that of males (33.9% vs 20.1%, respectively).

During the first wave, the proportion of patients who experienced pulmonary and extra-pulmonary complications during their hospital course was higher though without differences between females and males (Supplementary Table 3).

Kaplan-Meier survival curve shows sex-specific differences in the number of days spent in the ICU, with males requiring critical care for a longer time (30.8 ± 3.5 days in males vs 14.1 ± 1.7 days in females; log rank *P* < 0.001; Fig. 2B). Since females and males admitted to ICU statistically differ in age (males were older than their counterpart; Table 1), the variable age and wave were entered as covariate in the Cox model. Despite both age and waves were significantly associated with days spent in ICU (age: *P* = 0.001, HR = 0.976; CI = 0.966–0.986; wave: *P* = 0.007. HR = 1.620; CI = 1.141–2.300), denoting longer time in ICU for older individuals and longer time in ICU in the first wave compared with the second wave, the model having sex as predictor still remained highly significant (*P* = 0.014, HR = 1.589, CI = 1.096–2.303).

Kaplan-Meier survival curve also revealed a trend toward significance for a possible impact of sex on disease duration, suggesting a longer disease duration in males (30.1 ± 1.4 days) than in females (26.7 ± 1.3 days; log rank *P* = 0.056). When age and waves were entered as covariate in the Cox model, both age and wave were found to be significantly associated with disease duration (age: *P* = 0.001, HR = 0.982; CI = 0.977–0.987; wave: *P* = 0.001, HR = 0.706; CI = 0.602–0.827), denoting longer disease duration for older individuals and mainly in the first wave. In the Cox model, the predictor sex was significant (*P* = 0.021, HR = 1.210, CI = 1.030–1.422; Fig. 2C), denoting a longer disease duration for males rather than females. In addition, although the percentage of males who died in ICU was significantly higher, the total deaths’ rate among all patients included in the cohort was not significantly different between sexes (Table 1). While logistic regression analysis indicated that age has no influence of in the fatal cases of COVID-19 (Table 1), the mean age of deceased females was significantly higher (80.9 ± 10.3 years in females vs 72.2±9.6 years in males; *t* = 5.071, *P* = 0.000).

Logistic regression analysis, performed to evaluate the impact of comorbidities on clinical outcome (i.e., deaths), revealed that only cardiovascular disorders and neuropsychiatric conditions can be considered significant predictors of death. In fact, there were more deaths in patients with cardiovascular disorders than in those who did not (respectively 19.6% and 11.8% of mortality, *P* < 0.001). Similarly, deaths are more frequent in patients with neuropsychiatric conditions than in those without (respectively 27.5% and 12.9% mortality, *P* < 0.001). In contrast, endocrine diseases (*P* = 0.597), cancer/heme malignancy (*P* = 0.081), and autoimmune diseases (*P* = 0.249) did not emerge as significant predictors of death.

### Laboratory parameters of female and male patients hospitalized for COVID-19

Supplementary Table 4 reports the median and ranges found in both sexes and waves, of the highest values of these laboratory parameters, with the sole exception of platelets (PLT), of which the lowest values were identified. The number of white blood cells (WBC) and neutrophils, as well as the levels of high-sensitivity C-reactive protein (CRP) and fibrinogen were significantly more elevated in males than in females and in the first wave in comparison with the second one, and the effect of sex is stable along the two waves. Ferritin and alanine aminotransferase (ALT) were also higher in males, but constant in the two waves, while lactate dehydrogenase (LDH) levels were similar in females and males, but higher in patients hospitalized during the first wave. The lowest values of PLT were observed in males, in both waves. The number of patients with highest lymphocytes, monocytes, aspartate aminotransferase (AST), and procalcitonin values is equally represented in both sexes and waves.

**Fig. 3:**
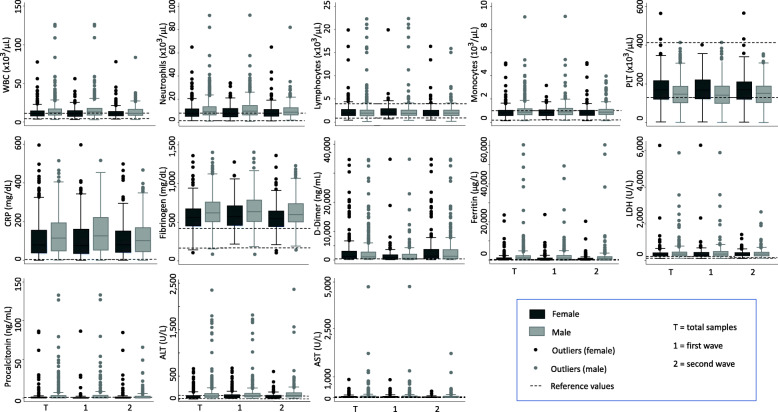
Sex distribution during the two waves of laboratory parameters of COVID-19 patients. The highest (the lowest for PLT) values identified in each patient during hospitalization are shown, together with the “outlier” and laboratory “reference” values. ALT, alanine aminotransferase; AST, aspartate aminotransferase; CRP, high-sensitivity C-reactive protein; LDH, lactate dehydrogenase; PLT, platelets; WBC, white blood cells

The highest levels of WBC, neutrophils, CRP, fibrinogen, D-dimer, ferritin, LDH, and procalcitonin were preferentially reported in patients admitted in ICU than in those that were managed in the other hospital units, or who died during the course of COVID-19 (Fig. 3, in which the laboratory data obtained in females and males were shown together with the “outlier” values and with the laboratory “reference” values, and Supplementary Table 5). Again, the lowest number of PLT was found in patients who did not survive.

We then checked whether the upper values of each parameter, identified as the furthest observations positioned within one and a half interquartile range of the upper end of the box, correlate with COVID-19 severity classes. Patients with COVID-19 and concomitant heme neoplasia had the most evident “outlier” blood cell count values and in particular males with critical disease. This feature was also observed for the other analytes; for instance, the total outlier values of CRP were found in critical male patients (vs 19.4% in females), which also had 83% of the outlier values of fibrinogen (vs 58% in females).

## Discussion

It is now well known, since the very first published studies, that the vast majority of COVID-19 deaths were among men, across all age groups, and that, overall, women dying for SARS-CoV-2 infection were older than men [21, 22]. This feature has been documented worldwide because a male bias in COVID-19 mortality was reported in the 37 out of the 38 countries that have provided sex-disaggregated data [8]. Other studies have also reported that a higher proportion of males were hospitalized than females [17, 23].

Our data, however, showed that this is not the case for younger females (< 45 years), who probably are more at risk than males of the same age of contracting COVID-19 as they account for the majority of healthcare workers, representing the first front-line during the COVID-19 outbreak [24, 25], and for older females (> 75 years), whose number of hospitalizations is almost double in the second wave compared to the first one. The similar number of hospitalizations of > 75 years old females and males could be due to the fact that in Brescia province the number of elderly women is about twice as many as that of men (49,472 vs 26,914) [26]. This, together with the greater attention paid to the elderly, due to the experience gained in the first months of the pandemic could explain the high number of women hospitalized in the second wave.

In our cohort, the mean time from symptom onset to hospitalization was 6 days, therefore within the range of 2.62 to 9.7 days reported in different countries [27], but there were not significant differences in the time between symptom onset and hospitalization in regard to sex and age groups, and waves. Although sex differences exist in the timeliness and extent to which patients seek healthcare in response to physical concerns [28], this did not occur in our patients’ cohort, probably due to the great danger and attention given to this disease.

In both waves, more males showed interstitial pneumonia, fever, and dyspnea at the time of hospitalization, while more females reported vomiting. An altered state of consciousness, equally reported by women and men and significantly more frequent in the first wave, was the principal no-COVID-19-related cause of hospitalization.

The presence of multiple comorbidities has been associated with a worse prognosis and higher mortality rate since the start of the COVID-19 pandemic [29]. As also reported by the most recent publications [17, 30], we confirmed that cardiovascular diseases and endocrinopathies, especially diabetes mellitus, are the most frequent comorbidities in patients admitted to our hospital, even though with no sex differences. This is in contrast with some recently published data obtained in 340 Italian and Spanish patients in whom cardiovascular disorders were more frequent in males [18]. Instead, our study confirms that asthma occurred more frequently in women [17, 30], together with psychiatric, musculoskeletal, and rheumatic disorders, and autoimmunity/immune-dysregulation diseases. Of interest, we found a correlation between the presence of multiple comorbidities and a worse prognosis, especially in regard to deaths. However, surprisingly, patients with the highest number of comorbidities are less frequently admitted in ICU. The most plausible explanation could be that the unavailability of ICU beds, especially during the peak of the two waves, might have influenced the access to ICU, favoring patients with higher survival chances. Another plausible reason could be that many multi-pathological patients died before the admission to ICU.

The most frequent comorbidities, such as cardiovascular disorders, that affected preferentially patients hospitalized in the second wave, and neuropsychiatric conditions appeared to significant predictors of death.

The complications occurred during the hospitalization were many, but equally distributed among both sexes and waves, with the sole exception of pneumothorax/pneumomediastinum cases which were less reported in the second wave, perhaps due to the accumulated experience of all clinicians in the use of non-invasive ventilation devices.

As it has already emerged worldwide that male patients have a high risk of requiring ICU admission [14], the rate of males admitted in ICU in our institution was higher than that of females. Males in ICU were older, had a longer ICU stay, and a longer disease duration, especially in the first wave and deceased more frequently in ICU than females. However, the rate of total deaths for hospitalized patients was not significantly different for sex and waves. This indicates that not only the proportion of females and males who test positive for SARS-CoV-2 and become ill with COVID-19 is the same [14], but also that the rate of hospital deaths is comparable, indicating that once hospitalized, the risk of dying is comparable in females and males. This occurs despite the many clinical characteristics of male patients, such as more hospitalizations, higher percentage of multi-symptomatic patients, more days spent in hospital and in ICU, and more ICU deaths appeared to be to their disadvantage. In addition, males also had the highest values of several laboratory biomarkers (i.e., WBC, neutrophils, CRP, fibrinogen, ferritin, and ALT), especially in the first wave (higher levels of D-dimer, in the second wave, were due to the improvement of the analytic method). These higher levels were found mainly in patients who required ICU management and then deceased. The high number of neutrophils but not of lymphocytes may come from the physiological responses of the innate immune system to systemic inflammation which is more intense in critical patients [31].

Thus, aging-related characteristics, which have been proposed to explain susceptibility to SARS-CoV-2 infection and progression to COVID-19 [32], could be the main reasons related to the fatal COVID-19 outcome observed in our cohort.

Many of the differences we have observed between the first and second wave were totally unexpected, also considering that no available data have been delivered on this aspect yet. One plausible reason could be the different approaches to the disease in the two waves, such as the patient management skills, the training and experience acquired by healthcare personnel, the increased number of beds reserved for COVID-19 patients, with dedicated staff and adequate equipment, as well as the scientific knowledge acquired on this newly discovered viral infection. The explanation cannot be the onset of viral variants that although present since August 2020 [33] have become the prevalent SARS-CoV-2 strain in Brescia only after February 2021. It might be interesting to investigate further patients hospitalized after this date.

The “limitations” of this report include the fact that our hospital, being one of the largest health facilities in Italy, is a reference center for some specific pathologies. As such, it is possible that hematologic patients, those with renal, neurological diseases, and immunodeficiency could be preferentially hospitalized in this facility. Furthermore, thanks to the large number of ICU beds, it is likely that the most serious cases are sent to our hospital as a referral point for the entire province and the neighboring ones. An important aspect, not investigated among our patients, is the therapy. However, an analysis of medications that were intended as therapeutics against COVID-19 revealed a similar pattern of usage between females and males for remdesivir, hydroxychloroquine, and steroids [17].

The “strength” of the study is that our cohort includes patients with a similar social context, the same access to care (because in Italy there is a free national health system, not based on private insurance, hence everyone has the same access to care and hospital admission), same ethnicity (almost all Caucasian), and enrolled within the same catchment area. In addition, all patients have been referred to a unique emergency department; therefore, patients have been hospitalized on the basis of the same characteristics and guidelines, and since hospitalized in the same hospital, they all had the same opportunity to access the same facilities and treatments.

## Perspectives and significance

Although males spent more days in hospital, had a more critical and longer duration of the disease, and were admitted and died in ICU more than females, the rate of deaths among all patients of our study did not show sex differences. Therefore, once patients were hospitalized, the risk of dying was similar between females and males. The biggest difference seems to be related to the number of admissions to the hospital, significantly higher for males. Therefore, future studies should aim at understanding the reason why despite being infected by SARS-CoV-2 in the same number of males, less females develop the disease requiring hospitalization.

## Conclusion

In conclusion, our findings provide a further piece in understanding the sex-related biases in COVID-19 and may provide an important basis for the development of a personalized approach to the treatment and care of female and male patients with COVID-19, including inclusion in clinical trials and vaccination.

## Supplementary Information

**Additional file 1: **Supplementary Table 1. Symptoms reported by COVID-19 patients at the hospital admission. Row numbers (percentages) and statistical significances, indicated as *P* value (chi square test), are reported for females and males, and for first and second wave. ^a^For the variable "Mean number of symptoms", the numbers denote mean (standard deviation) and statistical significance is indicated as *P* value (t test). The normal distribution of this variable was tested using the Kolmogorov-Smirnov test. The three columns at the right-hand side report the results of logistic regressions using sex and wave as predictors and age as covariate of no interest. A not significant sex x wave interaction denotes that the percentages of males and females for each condition do not differ across waves. Statistically significant results are reported in bold.

**Additional file 2:** Supplementary Table 2. Comorbidities affecting hospitalized COVID-19 patients. Row numbers (percentages) and statistical significances, indicated as *P* value (chi square test), are reported for females and males, first and second wave. ^a^For the variable "Mean number of comorbidities", the numbers denote mean (standard deviation) and statistical significance is indicated as *P* value (t test). The normal distribution of this variable was tested using the Kolmogorov-Smirnov test. The three columns at the right-hand side report the results of logistic regressions using sex and wave as predictors and age as covariate of no interest. A not significant sex x wave interaction denotes that the percentages of males and females for each condition do not differ across waves. Statistically significant results are reported in bold. COPD: chronic obstructive pulmonary disease, HIV: human immunodeficiency virus.

**Additional file 3:** Supplementary Table 3. Complications occurring to COVID-19 patients during hospitalization. Row numbers (percentages) and statistical significances, indicated as *P* value (chi square test), are reported for females and males, first and second wave. ^a^For the variable "Mean number of complications", the numbers denote mean (standard deviation) and statistical significance is indicated as *P* value (t test). The normal distribution of this variable was tested using the Kolmogorov-Smirnov test. The three columns at the right-hand side report the results of logistic regressions using sex and wave as predictors and age as covariate of no interest. A not significant sex x wave interaction denotes that the percentages of males and females for each condition do not differ across waves. Statistically significant results are reported in bold. ^b^Laboratory parameters not specifically associated with systemic inflammation and infections.

**Additional file 4:** Supplementary Table 4. Laboratory parameters of female and male COVID-19 patients who were hospitalized during the first and second pandemic wave. The table shows median and range values for females and males, first and second wave. Statistical significances are expressed as *P* value (ANOVA) and indicated in bold. The three columns at the right-hand side report the results of logistic regressions using sex and wave as predictors and age as covariate of no interest. ^a^Procalcitonin was tested in 455 patients. ALT: alanine aminotransferase, AST: aspartate aminotransferase, CRP: high-sensitivity C-reactive protein, LDH: lactate dehydrogenase, WBC: white blood cells.

**Additional file 5:** Supplementary Table 5. Laboratory parameters of patients who were admitted to ICU and who recovered or deceased. The table shows median and range values for patients who were not admitted to ICU (No ICU) and those who required ICU management; patients who survived (Alive) or deceased. Statistical significances are expressed as *P* value (t test) and indicated in bold. The three columns at the right-hand side report the results of logistic regressions using sex and wave as predictors and age as covariate of no interest. ALT: alanine aminotransferase, AST: aspartate aminotransferase, CRP: high-sensitivity C-reactive protein, ICU: intensive care unit, LDH: lactate dehydrogenase, WBC: white blood cells.

## Data Availability

The datasets used and/or analyzed during the current study are available from the corresponding author on reasonable request.

## Abbreviations

ALT: Alanine aminotransferase
AST: Aspartate aminotransferase
COVID-19: Coronavirus disease 2019
CRP: C-reactive protein
LDH: Lactate dehydrogenase
SARS-CoV-2: Severe acute respiratory syndrome coronavirus 2
WBC: White blood cells
