# Correction to: Sex differences in a cohort of COVID-19 Italian patients hospitalized during the first and second pandemic waves

**DOI:** 10.1186/s13293-021-00391-2

**Published:** 2021-08-20

**Authors:** Virginia Quaresima, Cristina Scarpazza, Alessandra Sottini, Chiara Fiorini, Simona Signorini, Ottavia Maria Delmonte, Liana Signorini, Eugenia Quiros-Roldan, Luisa Imberti

**Affiliations:** 1 Centro di Ricerca Emato-Oncologica AIL (CREA) and Diagnostic Department, ASST Spedali Civili of Brescia, Brescia, Italy; 2 Department of General Psychology, University of Padova, Padua, Italy; 3 University Department of Infectious and Tropical Diseases, University of Brescia, ASST Spedali Civili of Brescia, Brescia, Italy; 4 Immune Deficiency Genetics Section, Laboratory of Clinical Immunology and Microbiology (LCIM), National Institute of Allergy and Infectious Diseases (NIAID), National Institutes of Health (NIH), Bethesda, MD 20892-1456 USA

## Correction to: Biol Sex Differ 12:45 (2021) https://doi.org/10.1186/s13293-021-00386-z

Following publication of the original article [1], the authors reported a typesetting error in the citations in the Discussion section.

The following two paragraphs (paragraph 9 and 10 in the Discussion section) have been corrected to reflect citation [32, 33] respectively.

“Thus, aging-related characteristics, which have been proposed to explain susceptibility to SARS-CoV-2 infection and progression to COVID-19 [32], could be the main reasons related to the fatal COVID-19 outcome observed in our cohort.

Many of the differences we have observed between the first and second wave were totally unexpected, also considering that no available data have been delivered on this aspect yet. One plausible reason could be the different approaches to the disease in the two waves, such as the patient management skills, the training and experience acquired by healthcare personnel, the increased number of beds reserved for COVID-19 patients, with dedicated staff and adequate equipment, as well as the scientific knowledge acquired on this newly discovered viral infection. The explanation cannot be the onset of viral variants that although present since August 2020 [33] have become the prevalent SARS-CoV-2 strain in Brescia only after February 2021. It might be interesting to investigate further patients hospitalized after this date.”

The references can be accessed in the original article [1] which has been corrected.
